# Loading‐associated expression of TRIM72 and caveolin‐3 in antigravitational soleus muscle in mice

**DOI:** 10.14814/phy2.12259

**Published:** 2014-12-24

**Authors:** Yoshitaka Ohno, Takao Sugiura, Yoshinobu Ohira, Toshitada Yoshioka, Katsumasa Goto

**Affiliations:** 1 Laboratory of Physiology, School of Health Sciences, Toyohashi SOZO University, Toyohashi, 440‐8511, Japan; 2 Faculty of Education, Yamaguchi University, Yamaguchi, 753‐8513, Japan; 3 Faculty and Graduate School of Health and Sports Sciences, Doshisha University, Kyotanabe, 610‐0394, Japan; 4 Hirosaki Gakuin University, Hirosaki, 036‐8577, Japan; 5 Department of Physiology, Graduate School of Health Sciences, Toyohashi SOZO University, Toyohashi, 440‐8511, Japan

**Keywords:** Caveolin‐3, mechanical loading, skeletal muscle, tripartite motif‐containing 72

## Abstract

Effects of mechanical loading on the expression level of tripartite motif‐containing 72 (TRIM72) and caveolin‐3 (Cav‐3) in mouse soleus muscle were investigated. Mice were subjected to (1) continuous hindlimb suspension (HS) for 2 weeks followed by 1‐week ambulation recovery or (2) functional overloading (FO) on the soleus by cutting the distal tendons of the plantaris and gastrocnemius muscles. Soleus muscle atrophy was induced by 2‐week hindlimb suspension (HS). Reloading‐associated regrowth of atrophied soleus muscle was observed by 1‐week reloading following HS. HS also depressed the expression level of insulin receptor substrate‐1 (IRS‐1) mRNA, TRIM72, Cav‐3, and phosphorylated Akt (p‐Akt)/total Akt (t‐Akt), but increased the phosphorylated level of p38 mitogen‐activated protein kinase (p‐p38MAPK) in soleus muscle. Thereafter, the expression level of MyoD mRNA, TRIM72 (mRNA, and protein), and Cav‐3 was significantly increased and recovered to the basal level during 1‐week reloading after HS. Although IRS‐1 expression was also upregulated by reloading, the expression level was significantly lower than that before HS. Significant increase in p‐Akt and phosphorylated p70 S6 kinase (p‐p70S6K) was observed by 1‐day reloading. On the other hand, 1‐week functional overloading (FO) induced soleus muscle hypertrophy. In FO‐associated hypertrophied soleus muscle, the expression level of IRS‐1 mRNA, MyoD mRNA, TRIM72 mRNA, p‐Akt, and p‐p70S6K was increased, but the expression of Cav‐3 and p‐p38MAPK was decreased. FO had no effect on the protein expression level of TRIM72. These observations suggest that the loading‐associated upregulation of TRIM72 protein in skeletal muscle may depress the regrowth of atrophied muscle via a partial suppression of IRS‐1. In addition, downregulation of Cav‐3 in skeletal muscle may depress overloading‐induced muscle hypertrophy.

## Abstract (precis)

The mRNA expression level of MyoD and TRIM72 was significantly increased and recovered to the basal level during 1‐week reloading after unloading. Although IRS‐1 expression was also upregulated by reloading, the expression level was significantly lower than that before unloading. The loading‐associated upregulation of TRIM72 in skeletal muscle may depress the regrowth of atrophied muscle via a partial suppression of IRS‐1.

## Introduction

Skeletal muscle exhibits a large plasticity in response to changes in extracellular stimuli. Unloading as well as inactivity induces atrophy and weakness of skeletal muscle, especially in antigravitational soleus muscle (Riley et al. 1990; Caiozzo et al. 1994; Allen et al. 1996). On the contrary, mechanical loading, such as antigravitational activity as well as stretch, stimulates muscle protein synthesis and induces muscle hypertrophy (Goldspink 1999). It has been generally accepted that changes in skeletal muscle volume are attributed to changes in skeletal muscle cell volume with a constant of the number of skeletal muscle cells. Increase in skeletal muscle volume, so‐called muscle hypertrophy, occurs due to an imbalance between muscle protein synthesis and breakdown, with a resultant net gain in muscle protein (Goldspink et al. 1983). Akt and its downstream p70 S6 kinase (p70S6K), which stimulates muscle protein synthesis via increasing mRNA translation through the phosphorylation of ribosomal protein components, are suggested to be involved in muscle growth and hypertrophy in response to mechanical loading (Bodine et al. 2001; Latres et al. 2005; Sugiura et al. 2005; Wang and Proud 2006; Fujita et al. 2007; Sandri 2008). Even though the precise regulatory mechanisms for the volume of skeletal muscle cells are still unknown, it has been reported that expression of membrane‐related protein, such as transcription of tripartite motif‐containing 72 (TRIM72) and caveolin‐3 (Cav‐3), play a crucial role in myogenesis (Cai et al. 2009b; Lee et al. 2010).

TRIM72 is expressed in the plasma membrane and intracellular vesicles of skeletal and cardiac muscle cells, and is one of TRIM family composed of a really interesting new gene (RING) finger domain, a B‐box, two coiled coil (CC) domains, and a spla and ryanodine receptor (SPRY) domain (Cai et al. 2009a; Lee et al. 2010). It is well known that TRIM72 plays an important role in repair of skeletal muscle membrane (Cai et al. 2009a; Wang et al. 2010). TRIM72 is rapidly recruited to the injury site, and interacts with Cav‐3 to repair membrane damage (Cai et al. 2009a,b,c). Mice lacking TRIM72 are easily damaged by exercise, and develop a progressive myopathy with atrophy (Cai et al. 2009a). Recently, it has been also reported that TRIM72 is a negative regulator of myogenesis (Jung and Ko 2010; Lee et al. 2010). Although TRIM72 is transcriptionally upregulated by insulin receptor substrate‐1 (IRS‐1)/phosphatidylinositol 3‐kinase (PI3K)/Akt/MyoD pathway during myogenesis, TRIM72 inhibits IRS‐1 activation to regulate excess skeletal muscle differentiation and hypertrophy negatively (Jung and Ko 2010; Lee et al. 2010; Yi et al. 2013). Therefore, TRIM72 may be upregulated by mechanical loading that induces regrowth and hypertrophy of skeletal muscle.

Cav‐3, which is one of integral membrane proteins, is expressed exclusively in both cardiac and skeletal muscle, as well as smooth muscle cells (Glenney and Soppet 1992; Scherer et al. 1996; Song et al. 1996; Tang et al. 1996; Way and Parton 1996). It has been reported that the expression of Cav‐3 is upregulated by activation of Akt/p70S6K and p38 mitogen‐activated protein kinase (p38MAPK) pathways during myogenic differentiation (Galbiati et al. 1999; Fanzani et al. 2007). In addition, upregulation of Cav‐3 expression was also observed in hypertrophied fast muscles of insulin‐like growth factor (IGF) transgenic mice (Fanzani et al. 2007). Conversely, dexamethasone, which induces atrophic condition, downregulates Cav‐3 expression in C2C12 myotube (Fanzani et al. 2007). These observations suggest that the expression level of Cav‐3 may be upregulated in mechanical loading‐associated skeletal muscle hypertrophy. However, there is no report regarding the effects of loading on the expression of Cav‐3. In the present study, we investigated the expression level of TRIM72 and Cav‐3 in unloading‐associated atrophied and loading‐associated regrown, and hypertrophied skeletal muscle in mice. We also discuss a possible role of TRIM72 and Cav‐3 in loading‐associated regrown skeletal muscle.

## Materials and Methods

### Animals and grouping

All experimental procedures were carried out in accordance with the Guide for the Care and Use of Laboratory Animals of the National Institutes of Health (Bethesda, MD) and were approved by the Animal Use Committee of Toyohashi SOZO University. Male C57BL/6J mice, aged 11 weeks old were used (*n* = 40). All mice were housed in a home cage 20 × 31 cm and 13.5 cm height in a clean room controlled at approximately 23°C with a 12/12 h light–dark cycle. Solid diet and water were provided ad libitum.

### Experiment 1. Effects of hindlimb unloading followed by reloading

Five mice were assigned as the control, and were killed immediately before the initiation of hindlimb suspension (HS). Mice (*n* = 20) except the control were subjected to HS for 2 weeks. The HS of mice, which induces hindlimb unloading, was performed as following the methods described previously (Goto et al. 2004; Matsuba et al. 2009). Briefly, tails of the HS mice were cleaned, and were loosely surrounded by adhesive tapes cross‐sectionally, fixing a string at the dorsal side of the tail, to maintain the blood flow intact. The string was fastened to the roof of the cage at a height allowing the forelimbs to support the weight, yet preventing the hindlimbs from touching the floor and the sides of the cage. The mice could reach food and water freely by using their forelimbs. To induce reloading‐associated muscle regrowth, ambulation recovery was performed on fifteen mice immediately after the 2‐week HS.

### Experiment 2. Effects of functional overloading

Fifteen mice were used in this experiment. To induce overloading‐associated muscle hypertrophy, functional overloading (FO) on left soleus muscle was performed by cutting the distal tendons of the plantaris and gastrocnemius muscles under anesthesia with *i.p*. injection of sodium pentobarbital (50 mg/kg). Right soleus was assigned as a contralateral control.

### Sampling

In the experiment 1, soleus muscles were dissected from both hindlimbs before HS (Pre) and immediately (R0), 1 (R1), 3 (R3), and 7 (R7) days after HS (*n* = 5 in each day). In the experiment 2, soleus muscles were dissected from both hindlimbs 7, 14, and 21 days after the surgery (*n* = 5 in each day). Antigravitational soleus, but not plantaris and gastrocnemius, muscle was used in the present study because extensive investigations regarding the responses to various external stimuli have been performed by using soleus muscle (Kawano et al. 2004; Yasuhara et al. 2011). After the experimental period, the animals of each group were sacrificed by cervical dislocation. Then, both soleus muscles were excised from both hindlimbs. Dissected soleus muscles were rapidly weighed and frozen in isopentane cooled by liquid nitrogen. The samples were stored at −80°C until analyses.

### Western blotting and densitometry

Soleus muscles were cross‐sectionally cut into halves at the midbelly region. The distal portion of soleus was homogenized in isolation buffer (0.1 mL/mg muscle wet weight) of tissue lysis reagent (CelLytic^™^‐MT, Sigma‐Aldrich, St. Louis, MO) with 10% (v/v) Protease Inhibitor Cocktail (P8340, Sigma‐Aldrich) and 1% (v/v) Phosphatase Inhibitor Cocktail (524625, Calbiochem, San Diego, CA) and centrifuged at 12,000 rpm (4°C for 10 min), then the supernatant was collected. Protein concentration of the supernatant was determined by using the Bradford technique (protein assay kit; Bio‐Rad, Hercules, CA) and bovine serum albumin (Sigma‐Aldrich) as the standard. After the determination of protein concentration, the supernatant samples were mixed with sodium‐dodecylsulfate (SDS) sample buffer [30% (v/v) glycerol, 5% (v/v) 2‐mercaptoethanol, 2.3% (w/v) SDS, 62.5 mmol/L Tris‐HCl, 0.05% (w/v) bromophenol blue and pH 6.8] and were boiled for 3 min. The SDS‐polyacrylamide gel electrophoresis (PAGE) was carried out on 8 or 12% polyacrylamide containing 0.5% SDS at a constant current of 20 mA for 120 min. Equal amounts of protein (6 μg) were loaded on each gel. Molecular weight markers (Bio‐Rad Precision Markers) were applied to both sides of gel as the internal controls for transfer process or electrophoresis.

Following SDS‐PAGE, proteins were transferred to polyvinylidene difluoride membranes (Hybond‐P, GE Healthcare, Buckinghamshire, UK) using a Bio‐Rad mini transblot cell at a constant voltage of 100 V for 60 min at 4°C. After the transfer, the membranes were blocked for 1 h using a blocking buffer (RPN418, GE Healthcare). Then, the membranes were incubated for 2 h with a primary antibody: TRIM72 (PAB18940, Abnova, Taipei, Taiwan), Cav‐3 (ab2912, Abcam, Cambridge, UK), phosphorylated Akt (p‐Akt: 9271, Cell Signaling Technology Inc., Danvers, Mass.), total Akt (t‐Akt: 9272, Cell Signaling Technology Inc.), phosphorylated p70S6K (p‐p70S6K: 9205, Cell Signaling Technology Inc.), total p70S6K (t‐p70S6K: 9202, Cell Signaling Technology Inc.), phosphorylated p38MAPK (p‐p38MAPK: 9211, Cell Signaling Technology Inc.), total p38MAPK (t‐p38MAPK: 9212, Cell Signaling Technology Inc.), β‐actin (4967, Cell Signaling Technology Inc.), glyceraldehyde‐3‐phosphate dehydrogenase (GAPDH: G9545, Sigma‐Aldrich) and then reacted with a secondary antibody (anti‐goat or anti‐rabbit immunoglobulin G conjugate to horseradish peroxidase) for 2 h. After the final wash, protein bands were visualized using chemiluminescence (GE Healthcare), and signal density was measured by using Light‐Capture (AE‐6971) with CS Analyzer version 2.08b (ATTO Corporation, Tokyo, Japan). In the present study, the expression of β‐actin and GAPDH was evaluated to ensure the equal loading. In addition, the expression level of p‐Akt, p‐p70S6K, and p‐p38MAPK was evaluated by using the value relative to the expression level of t‐Akt, t‐p70S6K, and t‐p38MAPK, respectively. Each sample was run at least in duplicate on gels to ensure that results were not influenced by loading errors.

### Real‐time reverse transcription‐PCR

In the present study, the mRNA expression of MyoD, TRIM72, and IRS‐1 was assessed by real‐time reverse transcription‐polymerase chain reaction (RT‐PCR). Total RNA was extracted from the proximal portion of soleus muscle using the miRNeasy Mini kit (Qiagen, Hiden, Germany) according to the manufacturer's protocol. Samples (~10 ng of RNA) were reverse‐transcribed using the firststrand cDNA Synthesis kit according to the manufacturer's instructions [PrimeScript RT Master Mix (Perfect Real Time) for mRNA, Takara Bio, Otsu, Japan]. Synthesized cDNA was applied to real‐time RT‐PCR (Thermal Cycler Dice Real‐Time System II MRQ, Takara Bio) using Takara SYBR Premix Ex Taq II for mRNA, and analyzed with Takara Thermal Cycler Dice Real‐Time System Software Ver. 4.00 according to the manufacturer's instructions. The real‐time cycle conditions were 95°C for 30 sec followed by 40 cycles at 95°C for 5 sec and at 60°C for 30 sec for mRNA. To normalize the amount of total RNA present in each reaction, GAPDH was used as an internal standard.

The primers were designed by using the Takara Bio Perfect Real‐Time Support System (Takara Bio). Primers used for detection of mouse cDNA were as follows: MyoD, 5′‐ATTCCAACCCACAGAACCTTTGTC‐3′ (forward) and 5′‐TCAACCCAAGCCGTGAGAGTC‐3′ (reverse); TRIM72, 5′‐AAACCTGAGTTTGGGCCAAGAG‐3′ (forward) and 5′‐AGTGGATCGCAGGGCTGAA‐3′ (reverse); IRS‐1, 5′‐CATGATGGCTTGGCATTTGG‐3′ (forward) and 5′‐TGGCATAATGGTTAGTGCTGGAGA‐3′ (reverse); and glyceraldehyde‐3‐phosphate dehydrogenase (GAPDH), 5′‐TGTGTCCGTCGTGGATCTGA‐3′ (forward) and 5′‐TTGCTGTTGAAGTCGCAGGAG‐3′ (reverse).

### Statistical analysis

All values were expressed as means ± SEM. The comparison of data obtained from the experiments 1 was analyzed by using one‐way analysis of variance (ANOVA) for multiple comparisons followed by Tukey–Kramer test. Statistically significant level in the experiment 2 was analyzed by using a two‐way (time and treatment) ANOVA for multiple comparisons followed by Tukey–Kramer test. The significance level was accepted at *P* < 0.05.

## Results

### Effects of hindlimb unloading followed by reloading

**Figure 1.**
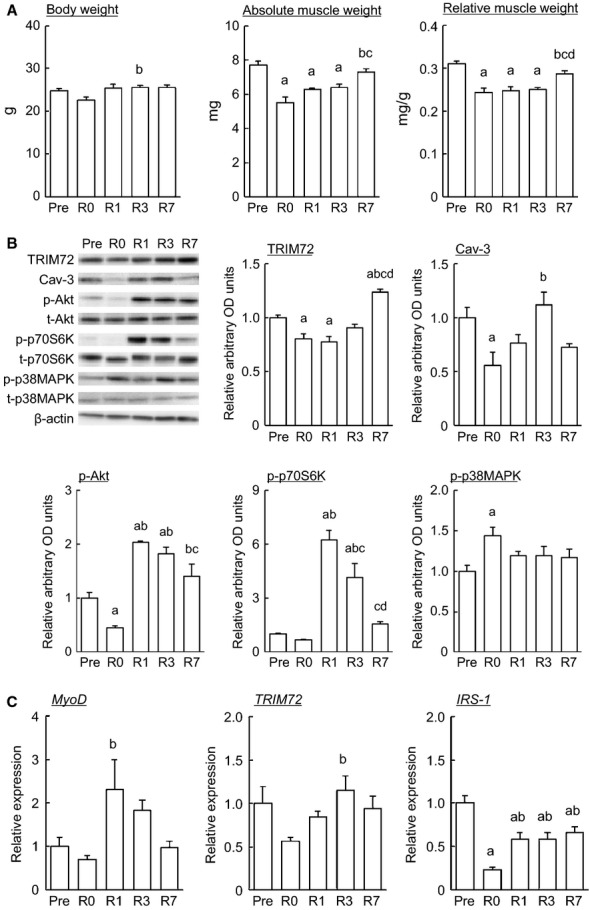
(A) Effects of hindlimb unloading followed by reloading on body weight and soleus muscle weight in mice. Relative muscle weight: muscle weight relative to body weight; Pre: before hindlimb suspension (HS); R0, R1, R3, and R7: immediately, 1, 3, and 7 days after HS, respectively. Means ± SEM. *n* = 5 in each group. a, b, c, and d: *P* < 0.05 versus Pre, R0, R1, and R3, respectively. (B) Representative expression pattern and mean expression level of TRIM72, Cav‐3, phosphorylated Akt/total Akt, phosphorylated p70S6K/total p70S6K, and phosphorylated p38MAPK/total p38MAPK in response to hindlimb unloading followed by reloading. TRIM72: tripartite motif‐containing 72; Cav‐3: caveolin‐3; p‐Akt: phosphorylated Akt; t‐Akt: total Akt; p‐p70S6K: phosphorylated p70 S6 kinase; t‐p70S6K: total p70S6K; p‐p38MAPK: phosphorylated p38 mitogen‐activated protein kinase; t‐p38MAPK: total p38MAPK. The value of p‐Akt, p‐p70S6K, and p‐p38MAPK is expressed as the relative expression of the phosphorylated forms to total expression forms. See Fig. 1A for other abbreviations, statistics, and symbols. Values are expressed relative to the value of Pre (1.0). Means ± SEM. *n* = 5 in each group. (C) Expression level of MyoD, TRIM72, and IRS‐1 mRNAs in response to hindlimb unloading followed by reloading. IRS‐1: insulin receptor substrate‐1. See Fig. 1A and B for other abbreviations, statistics, and symbols. Values are expressed relative to the value of Pre (1.0). Means ± SEM. *n* = 5 in each group.

Changes in body weight and soleus muscle weight in response to 2‐week HS followed by reloading were shown in Fig. 1A. There was a significant change in mouse body weight between R0 and R3 (*P* < 0.05). Two‐week HS significantly decreased absolute and relative muscle wet weights of soleus (*P* < 0.05). Thereafter, muscle weights showed a significant increase at R7 (*P* < 0.05), compared with the values at R0.

Changes in the protein expression level of TRIM72 and Cav‐3, and the phosphorylated level of Akt, p70S6K, and p38MAPK in response to 2‐week HS followed by reloading were shown in Fig. 1B. Protein expression level of both TRIM72 and Cav‐3 in the soleus was downregulated by 2‐week HS (*P* < 0.05). The expression level of TRIM72 in the soleus was gradually increased during 1‐week ambulation recovery following HS. A significantly increase in the expression level of TRIM72 was observed at R7 (*P* < 0.05). The expression level of Cav‐3 was gradually increased until 3 days of reloading. There was a significant difference in the expression level of Cav‐3 between R0 and R3 (*P* < 0.05). A significant decrease in p‐Akt expression was observed following 2‐week HS (*P* < 0.05), compared with the value at Pre. On the contrary, the expression level of p‐p38MAPK was significantly increased by 2‐week HS (*P* < 0.05), compared with the value at Pre. However, no significant change in p‐p70S6K expression was observed following 2‐week HS. One‐week ambulation recovery caused to increase in the expression of p‐Akt and p‐p70S6K (*P* < 0.05), compared with the value at Pre as well as R0. The higher expression level of p‐Akt and p‐p70S6K was maintained during ambulation recovery period.

Changes in the mRNA expression level of MyoD, TRIM72, and IRS‐1 in response to 2‐week HS followed by reloading were shown in Fig. 1C. Expression level of IRS‐1 mRNA was decreased by 2‐week HS (*P* < 0.05), but not MyoD and TRIM72 mRNA. Expression level of MyoD mRNA and TRIM72 mRNA was significantly upregulated by reloading following unloading (*P* < 0.05), and recovered to the level before unloading (Pre) during 1‐week reloading. Although IRS‐1 expression was also upregulated by reloading, ~30% suppression of IRS‐1 was observed compared to the basal level (Pre). Expression level of IRS‐1 during 1‐week reloading was significantly lower than that at Pre (*P* < 0.05).

### Effects of functional overloading

**Figure 2.**
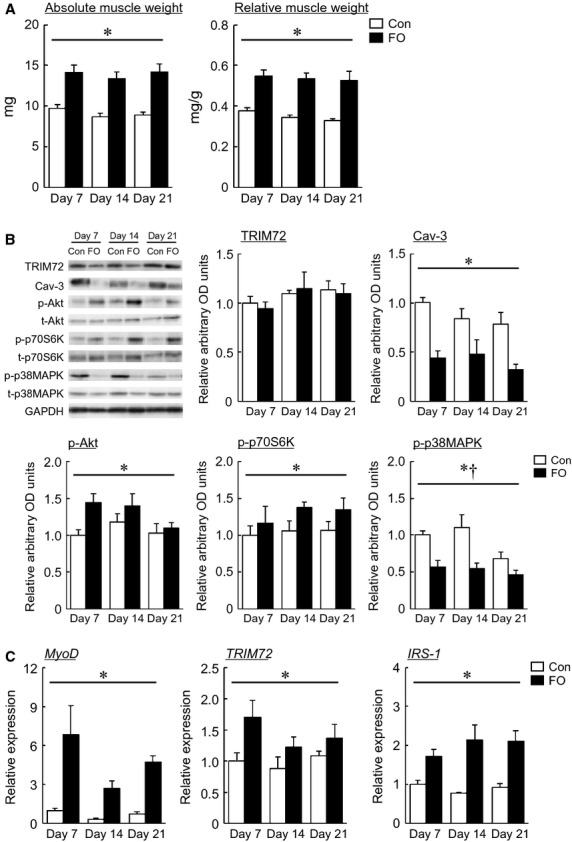
(A) Effects of functional overloading on soleus muscle weight in mice. Con: contralateral control; FO: functional overloaded. See Figure 1A for other abbreviations. Means ± SEM. *n* = 5 in each group. *Significant difference between Con and FO,* P* < 0.05. (B) Representative expression pattern and mean expression level of TRIM72, Cav‐3, phosphorylated Akt/total Akt, phosphorylated p70S6K/total p70S6K, and phosphorylated p38MAPK/total p38MAPK in response to functional overloading. GAPDH: glyceraldehyde‐3‐phosphate dehydrogenase. See Figs 1 and 2A for other abbreviations, statistics, and symbols. Values are expressed relative to the value of contralateral control at Day 7 (1.0). Means ± SEM. *n* = 5 in each group. ^†^Significant difference between Day 14 and Day 21, *P* < 0.05. (C) Expression level of MyoD, TRIM72, and IRS‐1 mRNAs in response to functional overloading. See Figs 1, 2A and B for other abbreviations, statistics, and symbols. Values are expressed relative to the value of contralateral control at Day 7 (1.0). Means ± SEM. *n* = 5 in each group.

Changes in soleus muscle weight in response to 3‐week FO were shown in Fig. 2A. There was no significant change in the body weight of mice by FO (data not shown). A significant main effect of treatment on the soleus muscle weight in mice was observed (*P* < 0.05). FO increased both the absolute and relative soleus muscle wet weights in mice (*P* < 0.05).

Changes in the protein expression level of TRIM72 and Cav‐3, and the phosphorylated level of Akt, p70S6K, and p38MAPK in response to 3‐week FO were shown in Fig. 2B. There were significant main effects of FO on the expression of Cav‐3, p‐Akt, p‐p70S6K, and p‐p38MAPK in soleus muscle (*P* < 0.05). FO significantly upregulated the expression level of p‐Akt and p‐p70S6K (*P* < 0.05). On the contrary, significant decrease in the expression of Cav‐3 and p‐p38MAPK was observed by FO (*P* < 0.05). There was a significant main effect of time on the expression of p‐p38MAPK (*P* < 0.05). A significant difference in p‐p38MAPK expression in the soleus was observed between Day 14 and Day 21 (*P* < 0.05). However, FO had no effect on the protein expression level of TRIM72.

Changes in the mRNA expression level of MyoD, TRIM72, and IRS‐1 in response to 3‐week FO were shown in Fig. 2C. There were significant main effects of FO on the mRNA expression of MyoD, TRIM72, and IRS‐1 in soleus muscle (*P* < 0.05). FO significantly upregulated the mRNA expression level of MyoD, TRIM72, and IRS‐1 (*P* < 0.05).

## Discussion

The present study demonstrated that 2‐week hindlimb unloading caused to downregulate the expression level of IRS‐1 mRNA, TRIM72, Cav‐3, and p‐Akt and to upregulate p‐p38MAPK expression in mouse soleus muscle. In addition, the mean expression of MyoD mRNA, TRIM72 (mRNA and protein), Cav‐3, p‐Akt, and p‐p70S6K in the soleus was upregulated by 1‐week reloading following unloading. Even though IRS‐1 expression was also upregulated by reloading, a partial suppression of IRS‐1 was observed. On the other hand, the expression level of IRS‐1 mRNA, MyoD mRNA, TRIM72 mRNA, p‐Akt, and p‐p70S6K in soleus muscle was increased by FO, but not TRIM72 protein expression. On the contrary, the expression of Cav‐3 and p‐p38MAPK was downregulated by FO.

### Soleus muscle mass and the expression of TRIM72 and Cav‐3

A significant reduction in soleus muscle weight was observed following 2‐week hindlimb unloading in the present study. This result is consistent with previously reported data from 2‐week hindlimb unloading in mice (Matsuba et al. 2009). It is generally considered that mechanical loading induces the regrowth of unloading‐associated atrophied skeletal muscle (Naito et al. 2000) as well as hypertrophy of normal muscle (Morioka et al. 2008). The present study demonstrated that the regrowth of atrophied soleus muscle was induced by reloading following 2‐week HS, and that muscle hypertrophy was induced by FO. These observations suggest that mechanical loading is a regulator of skeletal muscle mass.

In the present study, unloading‐associated decrease as well as reloading‐associated increase in the expression of TRIM72 (mRNA and protein) and Cav‐3 was observed in antigravitational soleus muscle. On the other hand, FO increased the expression of TRIM72 mRNA, but not the expression of TRIM72 protein, and decreased Cav‐3 expression in soleus muscle. This is the first report showing the profile of TRIM72 and Cav‐3 expressions in mature soleus muscle in response to unloading, reloading, and overloading in mice.

Since the expression level of TRIM72 and Cav‐3 affects C2C12 myogenesis (Cai et al. 2009b; Lee et al. 2010), the volume of mature skeletal muscle could be regulated by the expression level of both proteins. In the present study, reloading‐induced regrowth of atrophied soleus muscle was accompanied by the increase in TRIM72 and Cav‐3 protein expressions. However, the protein expression level of TRIM72 was not changed by FO that induced skeletal muscle hypertrophy. In addition, FO caused to decrease in the expression level of Cav‐3 protein. Therefore, the response of TRIM72 and Cav‐3 in reloading‐associated regrowth of atrophied soleus muscle was different from that in functionally overloaded muscle hypertrophy.

It has been suggested that insulin‐like growth factor (IGF)/IRS‐1/Akt/MyoD signaling upregulates transcription of TRIM72 during myogenesis, and that TRIM72 inhibits IGF‐induced IRS‐1 activation to regulate excess skeletal muscle differentiation and hypertrophy negatively (Jung and Ko 2010; Lee et al. 2010; Yi et al. 2013). In the present study, the expression level of p‐Akt, MyoD mRNA, and TRIM72 (mRNA and protein) recovered to the level before unloading (Pre) during 1‐week reloading following unloading. Although IRS‐1 expression was also upregulated by reloading, a significant suppression of IRS‐1 was observed compared to the basal level. Therefore, the suppression of IRS‐1 mRNA during reloading might be induced by upregulation of TRIM72 protein. On the other hand, the expression level of IRS‐1 mRNA, p‐Akt, MyoD mRNA, and TRIM72 mRNA was increased by FO, but not TRIM72 protein expression. FO‐associated regulation of IRS‐1 might be attributed to the stable expression level of TRIM72. In addition, upregulation of TRIM72 protein might play a negative feedback role to regulate reloading‐associated regrowth of atrophied muscle. However, additional experiments should be needed to elucidate TRIM72‐mediated regulatory mechanisms of skeletal muscle regrowth.

In C2C12 cells, the expression of Cav‐3 is also mediated by Akt signaling (Fanzani et al. 2007; Lee et al. 2010). Transfection of an activated form of Akt to C2C12 cells cause upregulation of Cav‐3 expression and muscle hypertrophy (Fanzani et al. 2007). In addition, drug‐induced atrophic condition downregulates Cav‐3 expression in C2C12 myotube (Fanzani et al. 2007). In the present study, unloading‐associated decrease in p‐Akt and Cav‐3 was observed in soleus muscle. Expression level of p‐Akt and Cav‐3 was increased by the reloading on unloading‐associated atrophied soleus muscle. On the other hand, Cav‐3 in soleus muscle was downregulated by FO, even though the expression level of p‐Akt was increased. In the overloading experiment, muscle weight (volume) was stable during from Day 7 to 21. Loading‐associated upregulation of Cav‐3 may be a negative regulator for muscle hypertrophy. These observations suggested that a possible role of TRIM72 and Cav‐3 in reloading‐associated regrown muscle might differ from that in overloading‐associated hypertrophy of skeletal muscle.

### p‐Akt, p‐p70S6K, and p‐p38MAPK

In the present study, the downregulation of p‐Akt in soleus muscle was induced by 2‐week hindlimb unloading. This result is consistent with the results from the previous studies in rat soleus (Sugiura et al. 2005) and gastrocnemius muscles (Haddad et al. 2006). On the other hand, the expression level of p‐Akt and p‐p70S6K in rat soleus muscle was increased by reloading following hindlimb unloading (Sugiura et al. 2005) and functional overloading (Spangenburg et al. 2008; Koya et al. 2013). In the present study, the expression of p‐Akt and p‐p70S6K in soleus muscle was also significantly increased by reloading following unloading as well as FO. As it is generally accepted that p70S6K, which is a downstream of Akt pathway, stimulates muscle protein synthesis via increasing mRNA translation through the phosphorylation of ribosomal protein components (Bodine et al. 2001; Latres et al. 2005; Sugiura et al. 2005; Wang and Proud 2006; Fujita et al. 2007), protein synthesis in loading‐associated regrowth of soleus muscle might be stimulated by the activation of Akt/p70S6K pathway.

In the present study, the expression level of p‐p38MAPK was increased by unloading, but was decreased by FO. It has been reported that p38MAPK in soleus muscle was activated by unloading (Morris et al. 2004; Hilder et al. 2005), and that p38MAPK stimulates ubiquitin ligase atrogin1/MAFbx and induces atrophy in response to muscle unloading (Morris et al. 2004; Li et al. 2005). Therefore, the activation of p38MAPK might negatively regulate skeletal muscle mass in response to loading.

In the present study, the expression level of t‐Akt, t‐p70S6K, and t‐p38MAPK in response to hindlimb unloading followed by reloading and FO was checked (Figure S1). Although there were no significant changes in t‐Akt and t‐p38MAPK in response to hindlimb unloading and reloading, a significant increase in the expression level of t‐p70S6K was observed (*P* < 0.05), compared with the value at R0. On the other hand, FO significantly upregulated the expression of t‐Akt and t‐p70S6K (*P* < 0.05), but not t‐p38MAPK. Even though the previous report also showed overloading‐associated increase in t‐Akt and t‐p70S6K in plantaris muscle (Marino et al. 2008), we have no clear explanation for the mechanical loading‐associated upregulation of t‐Akt and t‐p70S6K at present.

In conclusion, the response of TRIM72 and Cav‐3 in reloading‐associated regrowth of atrophied soleus muscle was different from that in functionally overloaded muscle hypertrophy. Upregulation of TRIM72 and downregulation of Cav‐3 in skeletal muscle may depress loading‐associated regrowth of atrophied muscle and overloading‐induced muscle hypertrophy, respectively.

## Acknowledgments

Authors thank Dr. L. L. Tang of Department of Physiology, Graduate School of Health Sciences, Toyohashi SOZO University for his technical assistance.

## Conflict of Interest

The authors have declared that no conflicts of interest exist.
